# Supplementary material for: Are carbon emissions trading and green financial instruments synergistic? -Comprehensive quantitative research based on content analysis
Source: PLoS One. 2024 Mar 7;19(3):e0298601. doi: 10.1371/journal.pone.0298601 (PMC10919601; doi:10.1371/journal.pone.0298601)
Supplement: S1 Data — (ZIP) [file pone.0298601.s001.zip › Data/地级市Z_F工作报告.zip!/╕ú╜¿/┴·╤╥╩╨/└┤╫╘╣1⁄2╓┌║┼í╛┬φ┐╦╩2╛▌═°í┐_2014.docx]

各位代表：

　　现在，我代表龙岩市人民政府向大会作政府工作报告，请予审议，并请市政协各位委员和其他列席的同志提出意见。

　　一、2013年工作回顾

　　过去的一年，面对严峻复杂的经济形势，在市委的正确领导下，全市各级政府坚持以邓小平理论、“三个代表”重要思想、科学发展观为指导，认真学习贯彻党的十八大、十八届三中全会和习近平总书记系列重要讲话精神，紧紧围绕主题主线，以提高经济增长质量和效益为中心，扎实开展“生态建设年”活动，坚定不移地推进结构调整、转型升级，全市经济社会发展稳中有进，较好完成了市四届人大常委会第十三次会议调整的目标任务。预计实现地区生产总值1500亿元，增长11.3%；公共财政总收入249.1亿元，增长5%；地方公共财政收入117.2亿元，增长15.5%；全社会固定资产投资1270亿元，增长27%；外贸出口总值21亿美元，与上年持平；实际利用外资2.2亿美元，增长8%；内联引资156亿元，增长10%；城镇居民人均可支配收入26150元，增长10%；农民人均纯收入10480元，增长11%；社会消费品零售总额492亿元，增长14%；居民消费价格总水平涨幅2.4%；城镇登记失业率2.5%；人口自然增长率8.4‰；年度节能减排目标预计可以实现。

　　一年来的主要工作和成效是：

　　（一）稳增长调结构深入推进

　　重点产业持续壮大。规模以上工业增加值488亿元，增长14.4%。机械和有色金属产业实现产值523亿元、增长26.5%，10个工业基地实现产值1206亿元、增长17.2%，环保、稀土、光电、新材料等产业加快发展。规模以上工业经济效益综合指数314%，居全省首位。新认定高新技术企业10家、累计59家。全年接待游客1780万人次，旅游总收入132亿元，分别增长20%和21%，永定荣获“中国旅游百强县”，梅花山旅游区成功创建国家生态旅游示范区。净增限额以上商贸企业396家、累计1312家。房地产以及信息、中介等现代服务业较快发展。

　　项目建设扎实推进。“五大战役”累计完成投资1200亿元，各战役投资完成比例居全省前列。一批重点项目加快推进，新龙马微型客车生产准入获国家工信部批准，古武高速武平段、湖城高速、新龙马微车、新罗龙化异地技改扩建一期、连城赛特新材二期等项目竣工投产；南三龙铁路、厦蓉高速龙岩段扩容工程、武平正德光电产业园、上杭泰山石膏系列产品、永定客家古镇、漳平木村工业园三期等项目开工建设。

　　开放协作深化拓展。建立市本级重大项目效益评估、市直部门利用外资工作绩效考评等制度，组建15个招商工作组，招商引资取得新成效。新对接“三维”项目148个，总投资767.2亿元。第四届海峡两岸机械产业博览会共签约项目51个，总投资377亿元。新引进2所民办学校、3家民营医院，民间投资占全社会固定资产投资的70%。山海协作进一步深化，新设2个共建产业园区。进出口总值首次突破30亿美元，连续三年入选中国外贸百强城市。对口援疆援藏深入推进。

　　政策扶持积极有力。研究出台帮扶工业、提升第三产业、发展现代农业、培育一县一优势产业等政策措施。组建市级融资性担保公司，新引进2家银行机构、2家股权交易运营中心，在全省率先实现村镇银行县域全覆盖。全市金融机构本外币存款余额1257.6亿元、贷款余额1179.1亿元，分别增长17.6%、13%。武平在全省率先实现林权直接抵押贷款。参照执行西部政策新增7项，累计97项、获得补助158.7亿元。争取项目用地指标位居全省前列、山区市首位。

　　（二）“三农”工作不断加强

　　农村经济稳步发展。农林牧渔业总产值281亿元、增长4.5%，粮食种植面积、单产和总产连续六年增长。茶叶、花卉苗木、林下经济等特色产业加快发展，武平成为首批“国家林下经济示范基地“。农产品加工业产值196亿元、增长21%，新增省级龙头企业9家、亿元以上企业13家。年出栏生猪千头以上、家禽万羽以上的设施养殖场分别达519家、267家。新发展农民专业合作社368家、累计1853家，发展家庭农场1084个。

　　基础设施进一步完善。农业综合开发继续走在全省前列，连续14年实现耕地占补平衡。流转耕地84万亩，流转比例达45%。水利建设投资22亿元，完成中小河流治理13条、病险水库除险加固32座，实施10个烟草水源工程。完成56.7万人农村饮水安全工程，“造福工程”和危房改造3.1万人。“三农”综合保险在全国率先实现全覆盖。新型职业农民培育工作创全国典型。启动“美丽乡村”建设试点，首批5个试点已基本建成。

　　（三）城镇建设步伐加快

　　中心城市建设扎实推进。中心城市规划体系更加完善，主城区建成面积扩大3平方公里，累计45平方公里。九大片区改造顺利推进，城北片区监狱搬迁改造获得国家司法部批准。双永高速北互通连接线、解放北路延伸段等项目基本完成，龙岩大道高架桥、城市大外环和一批城市综合体加快建设。新建4座人行天桥，新增绿地123.6公顷，石锣鼓湿地公园开园，莲花山栈道工程获全国人居环境范例奖。“两违”建设得到有效遏制，拆除违章建筑10.8万平方米。城市公交加快发展，环境卫生、道路交通整治成效明显。“三区两园”建设有力推进，“一主三新一卫”空间布局基本形成。

　　县域城镇加快发展。各县（市）城市建设力度加大，城市功能更加完善。15个省、市小城镇综合改革建设试点完成投资230亿元，占年度计划的175%。4个县（市、区）列入全国低丘缓坡地开发利用试点单位，新增规划建设用地指标3.3万亩。

　　（四）生态建设力度加大

　　“生态建设年”活动顺利开展。完成水土流失治理68.8万亩，占任务的120%。植树造林47.8万亩，占任务的119%，其中阔叶树37.8万亩，占造林总面积的79%。森林覆盖率由2011年的73.1%提高到74.3%，活立木蓄积量增加150万立方米，总量超过9000万立方米。重点流域水环境综合整治取得阶段性成效，拆除猪舍197万平方米，削减生猪存栏85万头，龙津河上游流域基本完成养猪场关闭退养，中心城区内河水质功能达标率54.2%、提高13.6个百分点，龙津河雁石桥省控断面水质13年来首次稳定在四类水标准。煤矿企业兼并重组、水源地保护区整治等工作扎实推进。在全省率先启动排污权交易工作。关闭落后产能企业42家，淘汰落后产能529.6万吨。中心城市空气环境质量优良率99.7%。成功创建省级森林城市，获评中国最具生态竞争力城市、全国生态文明建设试点城市。长汀成为全省首个国家水土保持生态文明县。全市获评省级以上生态乡镇119个、占总数的94.4%。

　　（五）民生保障更加有力

　　保障体系逐步健全。全面完成28项为民办实事项目。新增城镇就业2.4万人、农村劳动力转移就业5万人。保障性安居工程开工建设8548套、基本建成7188套。城乡居民社会养老保险制度实现全覆盖，农村居民大病保险实行市级统筹，城乡低保、被征地农民养老保障、城乡居民基本养老和新农合、城镇职工基本医疗保险等保障水平进一步提高。

　　社会事业协调发展。被评为全国科技进步先进市，7个县（市、区）全部通过科技进步考核，与清华大学共建龙岩紫荆创新研究院，国家级矿产品检测重点实验室基本建成。新罗区率先通过省级“教育强区”评估验收，义务教育标准化学校评估验收基本完成，启动实施百名“名校长”、千名“名师”培养工程，普通高考再次取得好成绩。基层医疗卫生综合改革走在全省前列，公立医院改革发展稳步推进。市第二医院新院、市中医院病房大楼、市第三医院门诊综合楼建成投入使用，有效防控H7N9禽流感疫情。全面完成广播电视“村村通”提升工程。成功举办第十四届市运会，我市运动员在第十二届全运会上勇夺5块金牌。公务员管理、编制、统计调查、民族宗教、外事侨务、老区扶贫、库区移民、食品药品、水文、气象、地震、地灾防治、人防、档案、地方志、科普、邮政、无线电管理、老年人、妇女儿童、青少年、残疾人以及慈善事业等工作取得新进展。

　　保持社会和谐稳定。“平安龙岩”建设扎实推进，有5个县（市）通过省级“平安先行县”考评验收，漳平被中央综治委授予“全国平安建设先进市”。依法治理和“六五”普法扎实开展。积极化解信访积案，社会矛盾纠纷排查调处机制进一步完善。安全生产形势持续稳定好转，事故起数、死亡人数连续8年下降。低生育水平持续稳定。“双拥”优抚扎实提升，龙岩市和7个县（市、区）均被评为省级双拥模范城（县）。

　　（六）自身建设持续强化

　　认真开展党的群众路线教育实践“先学先做先改”、“四下基层”、“马上就办”、“联乡挂村帮户”等活动。自觉接受市人大及其常委会的法律监督、工作监督和市政协的民主监督，广泛接受社会监督。办理人大代表建议315件、政协提案345件，办结率均达100%。行政服务网络向基层延伸，审批效率进一步提高。严格落实中央“八项规定”，进一步精简会议、文件、活动，削减“三公”经费支出。纠风专项治理和政风行风、机关效能建设扎实推进，廉政建设、行政监察和审计监督不断加强。深入开展电子政务和政府信息公开，建成全省最大的协同办公平台，被工信部确定为首批电子政务公共平台建设和应用试点示范市。

　　各位代表，成绩来之不易，这是市委正确领导的结果，是全市人民齐心协力、奋力拼搏的结果，是人大代表、政协委员和社会各界广泛参与、大力支持的结果。在此，我代表市人民政府，向全市人民，向人大代表、政协委员、各民主党派、工商联、各人民团体、无党派人士、离退休老同志和社会各界人士，向中央和省直驻岩机构、驻岩部队、武警官兵、公安民警，向关心支持龙岩发展的港澳台同胞、海外侨胞和国际友人，表示衷心的感谢！

　　在看到成绩的同时，我们也清醒地认识到存在的困难和问题，主要是：三次产业结构不尽合理，产业竞争力不强，转型升级压力大；城镇化率51%，比全省平均水平低8.6个百分点，中心城市聚集能力不强；农村基础仍然薄弱，农民持续增收难度较大；水土流失治理、水环境整治、节能减排、淘汰落后产能任务艰巨，生态文明建设任重道远；社会保障水平还比较低，部分群众生活还比较困难，安全生产、食品药品安全、社会治安还存在薄弱环节，社会治理面临新的挑战；部分政府工作人员服务意识不强，办事效率不高，有的单位官本位思想严重，“门难进、脸难看、事难办”，甚至存在不作为、乱作为和消极腐败现象。我们一定高度重视这些问题，采取有力措施切实加以解决。

　　二、扎实做好2014年工作

　　各位代表，今年是贯彻落实党的十八届三中全会精神、全面深化改革的重要一年。虽然我们面临不少困难和挑战，世界经济仍处在深度调整期，经济复苏存在不稳定不确定因素；与全国一样，我市经过多年高速增长后，进入增长速度换挡期、结构调整阵痛期、前期刺激政策消化期叠加的新阶段，烟草、建材等传统支柱产业增长乏力，黄金、煤炭等产业呈现下行态势，保持经济持续较快发展难度加大。但是，我们也要看到机遇和优势，发展仍处于大有作为的战略机遇期，改革红利将逐步释放，生态、文化、区位、老区等比较优势进一步显现，中央、省对苏区老区的支持力度不断加大，这些都将为我市发展注入新的动力和活力。我们要保持清醒，坚定信心，坚持以改革促发展、以改革促转型、以改革惠民生，把握机遇，奋力攻坚，推动闽西老区各项事业迈上新台阶。

　　政府工作的总体要求是：以邓小平理论、“三个代表”重要思想、科学发展观为指导，深入贯彻落实党的十八大、十八届三中全会和习近平总书记系列重要讲话精神，坚持稳中求进工作总基调，进一步解放思想、改革创新，着力产业发展，着力城乡建设，着力生态文明建设，着力保障和改善民生，走一条“绿色经济·生态家园”的科学发展之路，加快建设美丽、开放、幸福的全国生态文明建设先行区、国家可持续发展实验区、闽粤赣边区域性中心城市。

　　今年经济社会发展的主要预期目标是：地区生产总值增长11%；地方公共财政收入增长11%；全社会固定资产投资增长22%；外贸出口增长7%，实际利用外资增长5%；城镇登记失业率控制在4.2%以内；社会消费品零售总额增长14%；居民消费价格总水平涨幅3.5%左右；城镇居民人均可支配收入增长10.5%，农民人均纯收入增长11%；完成年度节能减排任务。

　　实现上述目标，重点抓好以下七个方面工作：

　　（一）推动工业加快转型升级

　　以龙头带动产业提升。坚持抓龙头、铸链条、建集群，注重存量提升和增量优选，增强产业实力和竞争力。实施“产业龙头促进计划”，加大企业扶持力度，争取新增规模企业100家。以龙工、龙净、新龙马、紫金矿业、金龙稀土等企业为龙头，大力引进实施关联配套项目，不断延伸产业链条，推动机械、有色金属等优势产业聚集发展，壮大产业集群。培育引进龙头企业，推动光电、新材料、新能源、节能环保、生物医药等战略性新兴产业加快发展。鼓励企业重组改造，推动烟草、建材、煤炭、钢铁、纺织等传统产业加快调整升级。有序推进中心城区工业企业退城进园，异地改造提升。推进“一县一优势产业“发展，加快培育领军企业，做大特色支柱产业。

　　以园区吸引产业聚集。创新开发区、工业园区管理及投融资机制，加快推进产品质量监督检测等平台建设，完善物流配送、科研创新、金融服务、信息咨询等配套服务体系。促进龙岩经济技术开发区与县（市、区）、新区合作共建园区，着力打造永丰、龙雁及各县（市、区）的工业发展平台，培育做大汽车、稀土以及金铜、硬质合金、不锈钢、光电等专业园区，规划建设信息产业园。

　　以项目促进产业扩张。开展产业转型升级、宜居环境建设、城乡基础设施提升、现代农业加快发展和民生工程“五大战役”重点行动计划。实施重点项目建设“223行动计划”，即抓好206个省、市重点项目，总投资2026亿元，年度投资302亿元，实现重点项目数、总投资、年度计划投资“三个增长”。突出抓好30个总投资超过亿元的重大工业项目、100个重点工业技改项目、200个重点工业新增长点项目，力争完成工业固定资产投资700亿元，新竣工、新投产工业项目200个，规模以上工业增加值增长13.5%以上。力争新龙马发动机、武平正德光电、上杭泰山石膏纸面石膏板、长汀中石油催化剂、漳平亿统机械等项目竣工投产，新龙马卡车扩建、新罗龙泰新能源、长汀清华紫荆稀土材料和永磁电机、连城赛特新材三期等项目开工建设。大力支持现有企业实施技改、扩建项目，进一步做大做强。

　　以创新推动产业提质。强化科研市场导向，突出企业创新主体地位，切实增强科技创新能力。“以升促建”推动高新技术产业开发区建设，加快科技创业园二期、海西科技创业城、紫荆创新研究院和院士工作站、企业博士后科研工作站等公共技术服务平台建设，建立重大科研设备仪器共享机制，力争培育省级以上新产品10个。实施高新技术企业成长助推计划、“才智龙岩”高端人才聚集计划，设立青年科技人才及科技创新创业人才培养专项资金，激发社会创新活力。

　　（二）大力提升农业现代化水平

　　高度重视粮食生产。坚持工业反哺农业、城市支持农村和多予少取放活的方针，全面落实强农惠农富农政策。完善种粮补贴政策，稳定粮食生产，开展高产创建活动，提高粮食单产和优质率。加强省级粮食产能区和粮库建设，改造提升粮食专业批发市场。

　　加快发展设施农业。推进果蔬、畜禽、食用菌、渔业设施化，做大做强花卉苗木、茶叶、竹业、肉鸡等特色产业，培育发展林下经济和观光休闲农业。提升国家级现代农业示范区、台湾农民创业园、福建农民创业园及示范基地建设水平，引导农业企业、精品项目向园区聚集。力争今年新增全国休闲农业与乡村旅游示范县1个，省级休闲农业示范乡镇5个。推进连片土地整治、农田改造，实施12个烟草水源工程、29个中小型水库除险加固、15条重点中小河流治理和九龙江北溪防洪工程建设，完成农田基本建设10.5万亩，夯实设施农业发展基础。

　　构建新型农业经营体系。继续实施“亿元企业计划”，培育农产品加工领军型龙头企业，扶持农产品营销大户和营销企业，力争农产品加工产值增长15%以上。规范提升农民专业合作社，大力扶持家庭农场、专业大户等新型主体。抓好新型职业农民培育工作，力争在全省率先实现全部县（市、区）列入全国试点县。鼓励和引导工商资本发展现代种养业。

　　切实保障农产品质量安全。抓好“菜篮子”工程，力争新建无公害蔬菜生产基地1000亩，无公害蛋禽基地新增存栏10万羽。健全市县乡农产品质量安全检测监管体系，加强重大动植物疫病防控，扩大农产品生产可追溯试点建设，推进农产品从田头到餐桌的全过程监管。

　　（三）扎实推进新型城镇化

　　着力提升规划水平。健全规划管理体制，加强城市空间开发利用管制，进一步提升规划的科学性、前瞻性、系统性和权威性。制定实施新型城镇化规划，科学确定中心城市和县域城镇的功能定位、规模形态、开发边界，优化城镇布局。切实保护好现有山水田林，延续历史文脉，彰显地方特色，让居民望得见山、看得见水、记得住乡愁。

　　加快城镇建设。围绕打造闽粤赣边区域性中心城市，稳步推进十大片区改造，高标准开发建设城北、城东片区，推进火车站北站房及城市综合体等重点项目建设；加快绿道、慢道、人行天桥、排水排污以及菜市场、加油加气站等基础设施建设，力争新增2000个停车位，确保登高山与莲花山连接栈道桥建成投入使用；落实公交优先战略，加强公交首末站、综合枢纽建设，新增公交车50台。扎实推进新区园区建设，推动公共服务体系向新区延伸。统筹推进县域城市产业、商贸、人居和基本公共服务项目建设，促进县城扩容提质。扎实推进“321”特色乡镇建设，抓好15个省、市小城镇综合改革建设试点。

　　推进宜居环境建设。深入开展城乡环境综合整治“点线面”攻坚计划，抓好“三边三节点”整治和棚户区改造。力争整治公路铁路沿线60公里以上，完成龙厦铁路沿线景观整治。加强重点流域、重点景区污水处理设施建设。在保留村庄原始风貌的基础上，稳步推进村庄整理、住宅建设，重点整治旧房裸房、生活环境和农村污水，突出抓好34个市级和140个县级“美丽乡村”试点建设，打造美丽村庄。坚持部门联动、依法依规、疏堵结合、源头防范，扎实开展“两违”综合治理专项行动，全面遏制违法占地、违法建设行为，建立城乡环境管护长效机制。

　　完善城乡基础设施。实施城乡基础设施建设“六项提升工程”，力争今年完成投资300亿元以上。抓好赣龙铁路扩能工程、南三龙铁路、漳永高速、古武高速武平至江西段、厦蓉高速龙岩段扩容工程、厦蓉高速龙岩互通迁建工程等项目建设；推进浦建龙梅、长泉铁路、冠豸山机场扩能改造等项目前期工作。抓好中心城市外环路、龙岩大道高架桥建设，开工建设华莲西路延伸段，推进龙腾路南延前期工作。抓好3条快速通道和省道308线红尖山隧道建设，力争中心城区通往永丰新区快速通道建成通车。实施公路安保工程1200公里，力争农村通客车率达98%，完善城乡客运服务体系。健全农村道路、水利设施管理养护机制。实施新一轮农网改造升级工程，新建扩建4座220千伏变电站、5个110千伏输变电工程。力争西气东输龙岩段竣工投产，推进海西天然气管网二期工程，规划建设天然气支线管网，做好气源对接和设施联通。加强“数字龙岩智慧城市”建设，促进信息共享和集成应用。

　　有序推进农业转移人口市民化。按照自愿、分类、有序的要求，促进有能力在城镇稳定就业和生活的常住人口实现市民化。推动教育、医疗等基本公共服务由户籍人口向常住人口覆盖，将符合条件的流动人口逐步纳入城镇住房和社会保障体系，在农村参加的养老保险和医疗保险规范接入城镇社保体系。力争全市城镇化率提高1.5个百分点以上。

　　（四）加快发展第三产业

　　大力发展现代流通业。继续实施商贸流通业“十百千工程”，新培育限上企业100家、龙头骨干企业10家。积极引进国内外品牌企业，加快商贸项目建设，优化消费环境，做大商贸流通业。扎实推进中国（龙岩）农产品物流交易中心、闽西汽贸交易城、闽西建材交易城等项目建设，推动物流业与制造业联动发展，打造现代物流基地。抓好交通运输、仓储等产业的改造升级。

　　做特做优旅游业。抓好省级旅游综合改革试点，积极推进国家智慧旅游试点城市建设。加快精品景区建设，重点抓好梅花山生态旅游示范区和永定客家文化旅游创意产业园、新罗花漾江山旅游综合体、梁野山旅游综合开发等项目建设，争取龙岩旅游集散中心投入运营。启动连城冠豸山创建国家5A级景区工作，力争古田景区创建5A级旅游景区。加大红色、客家、生态旅游资源整合力度，完善旅游要素，加强体验性旅游项目开发，扩大旅游消费。深化旅游统一营销，积极拓展动车沿线客源市场。推进旅游服务标准化建设。

　　培育发展新兴服务业。鼓励企业集团总部、交易平台、金融保险等机构进驻龙岩，发展总部经济、楼宇经济。支持建设特色电子商务平台，培育做大网上零售企业。加快发展医疗保健、养老休闲、体育健身以及会计、信息、咨询、中介及其它服务业，培育会展经济。加强房地产市场监管。培育引进优质物业公司。

　　（五）深入推进生态文明建设

　　抓好重点整治。统筹推进治山、治水、治大气，提高治理实效。总结提升“长汀经验”，扎实抓好新一轮水土流失治理，完成54.8万亩治理任务。推进重点流域综合整治，严格水环境监管，力争九龙江流域、中心城区内河水质功能区达标率分别达到90%和60%，闽江连城段、汀江流域水质达标率分别稳定在90%和95％以上。加强养殖业污染治理，推进生猪养殖减量提质，完成禁养区、限养区划定工作。推进饮用水源一、二级保护区的群众搬迁和环境整治，完成黄岗水库水源地整治阶段性任务。推进煤矿整顿关闭，加强矿区整治和生态恢复。强化重金属污染防治和造纸、印染企业污染治理。推进重点行业脱硫、脱硝、除尘改造，实施建筑施工扬尘控制、机动车尾气污染治理，开展中心城市PM2.5监测。

　　强化节能减排。完善节能激励约束机制，抓好30个重点节能与循环经济项目建设，推进上杭蛟洋工业集中区、龙雁经济开发区争创国家级循环经济示范园，确保重点用能单位节能16万吨标准煤。完成水泥、铁合金、煤炭等行业年度淘汰落后产能任务。

　　提升生态质量。围绕推进全国生态文明建设试点和创建国家环保模范城市、国家森林城市、全国绿化模范市，编制生态文明建设规划，科学划定生态红线区域，强化生态功能区保护。坚持生态保护与科学开发并举，大力发展生态经济，构建汀江生态经济走廊，促进“百姓富”与“生态美”的有机统一。深入推进“四绿”工程，造林绿化11.9万亩，其中阔叶树占70%以上，森林抚育14.5万亩，抓好679万亩重要生态区域的封山育林工作。力争通过省级生态市验收，长汀、上杭通过国家级生态县验收，新罗、永定、武平通过省级生态县（区）验收。

　　（六）更加积极扩大开放

　　发展对外经贸。完善外贸扶持政策措施，推进通关便利化，用好陆地港、加工贸易梯度转移重点承接地等平台，壮大出口主体和规模。优化出口结构，支持企业开展自营出口，扩大地产品出口总量。鼓励有条件的企业对外投资合作。

　　拓展区域合作。办好海峡两岸机械产业博览会，提升漳平台湾农民创业园建设水平，推进与台湾精密机械、光电信息、精细农业、旅游等产业深度对接。加强海峡两岸交流基地建设，深化龙台文化教育、医疗卫生等领域的交流合作。加强与港澳侨合作，提升对外交流水平。加快推进山海协作共建产业园，积极推动与赣州共建产业园区，大力发展“飞地”工业，承接沿海产业梯度转移。继续抓好对口援疆援藏工作。

　　提高招商实效。坚持一手抓新项目的引进，一手抓签约项目的落地建设。整合招商资源，健全招商联动机制，推行产业招商、园区招商、以商引商、小分队招商，提高招商工作实效。盯住世界500强企业、全国民企500强企业、央属企业，加大岩资“回归工程”实施力度，策划、引进一批重大项目。坚持选商选资，严把项目质量关。

　　（七）着力保障和改善民生

　　办好惠民实事。着力解决关系群众切身利益的突出问题，抓好20项为民办实事项目，促进改革发展成果更多惠及全市人民。

　　推进扶贫开发。重点推进长汀、连城、武平3个省级扶贫开发工作重点县、31个市级重点乡（镇）和320个贫困村扶贫工作。继续实施扶贫开发整村推进，加强产业扶贫、智力扶贫，增强贫困地区的“造血”功能。实施“造福工程”和危房改造3万人，新建46个省、市集中安置区。加大革命基点村和少数民族乡村帮扶力度，深入推进老区村跨越发展工程。争取国家和省里支持设立长（汀）连（城）武（平）扶贫开发试验区，在产业发展、开放合作、管理体制、投融资等方面先行先试。

　　健全社会保障。完善就业创业服务体系，新增城镇就业2.2万人、农村劳动力转移就业4.5万人。继续提高城乡低保、农村五保补助标准，健全临时救助制度，兜住困难群众基本生活底线。完善城乡居民社会养老保险一体化制度，推进城乡居民医疗保险一体化。完成40万人农村饮水安全工程建设。开工建设保障性安居工程1.2万套，基本建成1.1万套。加快发展老年人事业，提高居家养老服务水平。完善未成年人、农村留守儿童、妇女、残疾人关爱服务体系，积极发展慈善事业。

　　提高教育质量。统筹城乡义务教育资源均衡配置，深化“教育强县”创建工作。加快推进城区中小学扩容，支持名校办分校、老校带新校、强校扶弱校，扩大优质教育资源覆盖面。新建、改扩建公办幼儿园25所，加快龙岩一中分校、厦门大学龙岩附属学校等中小学建设，争取龙岩外国语学校秋季招生开学。推进龙岩大学、龙岩卫生职业学院筹建工作，支持闽西职业技术学院创建国家骨干高职院校。

　　加强健康服务。扩充医疗卫生资源总量，加强紧缺学科建设，力争市第一医院门诊楼、人民医院病房综合大楼建成投入使用，积极推进市第一医院新院前期工作。深入推进公立医院综合改革，加强公共卫生服务体系建设，着力办好农村卫生院和社区卫生服务中心。积极引进有实力的民办医疗机构，促进多元办医。积极开展全民健身活动，提高竞技体育水平，加快推进市老年人体育活动中心和海峡两岸（龙岩）体育交流基地等项目建设。对接完善生育政策，稳定低生育水平，促进人口长期均衡发展。加强食品药品安全监管。

　　发展特色文化。深入挖掘红色文化、客家文化、闽南文化内涵，打造文化精品。继续实施文化惠民工程，完善文化基础设施。加强历史文化名城、名镇、名村、名街和历史文物、非物质文化遗产保护。着力提升“海峡客家”品牌，争取创建国家级闽西客家文化生态保护试验区。加大文化市场主体培育力度，推动文化与旅游、科技融合，发展文化创意产业，提升文化产业规模化、专业化水平。积极推进龙岩文化园规划建设。

　　推进社会治理创新。深入开展“一评估四联动”，有效化解社会矛盾。畅通群众诉求表达渠道，推行信访听证和网络信访，维护信访秩序。深化“平安龙岩”建设，推进“平安和谐乡镇（村居）”创建工作，完善立体化社会治安防控体系，提升打防管控水平，有效防范和坚决打击各类违法犯罪活动，维护社会安定有序。加快建设全市信用信息平台，建立健全守信激励、失信惩戒联动机制。健全安全生产责任体系，强化安全隐患排查治理，扎实推进道路交通安全综合整治和打非治违专项行动，深入开展安全生产标准化建设，坚决遏制重特大安全事故。完善防汛抗旱和森林防火工作机制，增强防灾减灾能力。抓好第三次经济普查。深化军民融合，加强国防动员和后备力量建设，落实优抚安置政策，扎实推进新一届全国“双拥模范城（县）”创建工作。

　　三、全面深化改革，增强发展的动力和活力

　　万事开头难，知难就不难。我们将以一往无前的精神，深入贯彻中央、省和市委全面深化改革部署，落实年度改革任务，努力在重点领域和关键环节取得突破，让今天的改革为明天的发展铺路。

　　加大简政放权力度。完成市、县两级政府机构改革，进一步理顺市区、部门、社区职责。推进事业单位分类改革，普遍推行竞聘上岗制度。全面清理市、县两级行政审批事项，进一步下放审批权限，优化审批流程，推行行政审批全程网上办理。推广行政服务标准化，加强审批服务和事中事后监管。推进行业协会、商会与行政机关脱钩，促进社会组织规范发展、依法自治、发挥作用。完善政府购买社会服务制度。强化预算约束和财政支出绩效评价，开展部门预算绩效管理和监督。规范政府性债务管理，健全债务偿还机制。

　　充分激发市场主体活力。按照使市场在资源配置中起决定性作用和更好发挥政府作用的要求，正确处理政府与市场关系。研究制定市场准入负面清单，支持各类市场主体依法平等进入市场。全面推进工商登记制度便利化，营造规范统一、宽进严管的市场环境。组建公共资源交易平台，推动公共资源市场化配置项目进场集中交易。鼓励和引导民间资本及各类产业投资基金投资战略性新兴产业、基础产业和社会事业。

　　促进要素资源科学有效配置。推进“引银入岩”，完善担保体系，建立中小企业直接债务融资发展基金和风险补偿资金，优化金融生态环境。完善政银企合作、中小企业公共服务平台，加强增信服务和融资支持。用好“6·18”、“9·8”、省民企产业洽谈会等平台，促进企业技术、人才和项目、产业对接。深化集体林权制度改革，探索林权公开流转机制，盘活林农林木资产。抓好漳平国家级土地承包经营权确权登记颁证试点。加快建立土地流转准入、风险评估和监督保障制度，促进农村闲置土地合理利用。开展流域生态补偿机制研究和试点，健全矿产资源有偿使用制度。

　　四、加快建设法治政府和服务型政府

　　信任就是责任，重托饱含期待。我们将以开展党的群众路线教育实践活动为契机，大力弘扬古田会议精神、才溪乡调查精神，始终坚持为民、务实、清廉，不断加强政府自身建设。

　　突出依法行政。强化法治思维、法治意识，把依法行政贯穿于政府工作各个环节。坚持用制度管权管事管人，确保权力公开透明、规范运行，促进社会公平正义。自觉接受人大及其常委会的法律监督、工作监督和政协的民主监督，主动接受舆论监督，强化政府内部监督，增强政府执行力、公信力。认真办理人大代表建议、政协提案，提高办理质量。

　　突出务实为民。始终保持奋发向上、开拓进取的锐气，敢于迎难而上，奋力攻坚克难。坚持一切为了群众，一切依靠群众，深入开展“四下基层”、“联乡挂村帮户”活动，尊重实践，尊重规律，实干实效。树立正确的权力观和政绩观，着力谋长远打基础，多做对龙岩人民、对子孙后代、对社会有益的事。

　　突出廉洁高效。严格执行中央“八项规定”，切实解决“四风”方面存在的突出问题。发扬艰苦奋斗的优良传统，厉行节约反对浪费，严控“三公”经费，集中财力保发展、保民生。加强绩效管理，深入开展“马上就办”活动，不断提高行政效能。严格落实廉政准则和党风廉政建设责任制，从严查处违法违纪案件，做到干部清正、政府清廉。

　　各位代表，让我们紧密团结在以习近平同志为总书记的党中央周围，在市委的领导下，进一步解放思想，改革创新，同舟共济，勇于担当，为在更高起点上推进闽西老区科学发展、跨越发展、加快崛起而努力奋斗！
